# Supplementary figures and images for: Five new Caenorhabditis species from Indonesia provide exceptions to Haldane’s rule and partial fertility of interspecific hybrids
Source: bioRxiv. 2025 May 16:2025.05.14.653126. Preprint. [Version 1] doi: 10.1101/2025.05.14.653126 (PMC12132545; doi:10.1101/2025.05.14.653126)

*C. elegans* HPT48

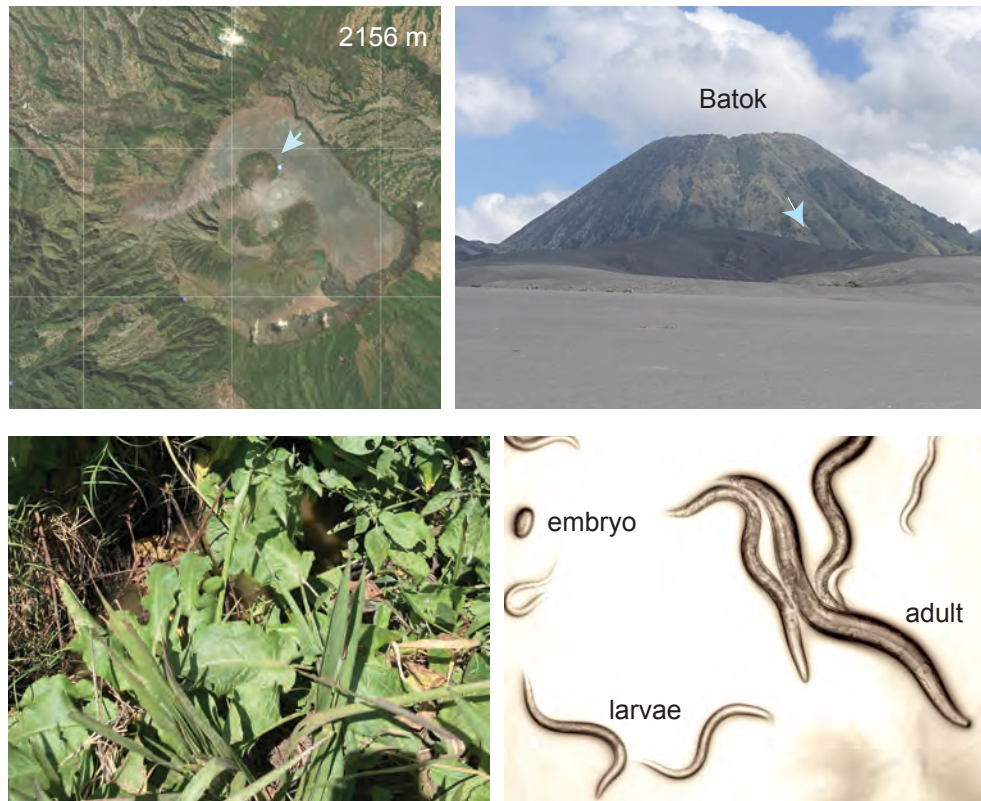

Figure S1

Supplement: Supplement 1 — Figure S1. Location and sample yielding C. elegans. For the C. elegans isolate, the pictures show the sampling site location at the bottom of the Batok and Bromo volcanoes, the decomposing stems and the laboratory culture (HPT48). [file media-1.pdf]

*C. indonesiana* HPT10

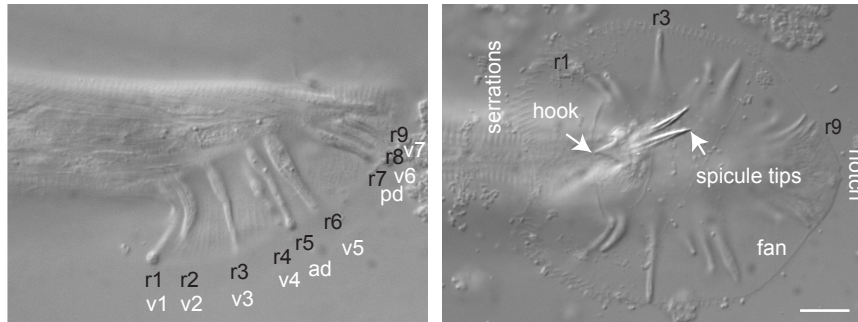

*C. malinoi* HPT35

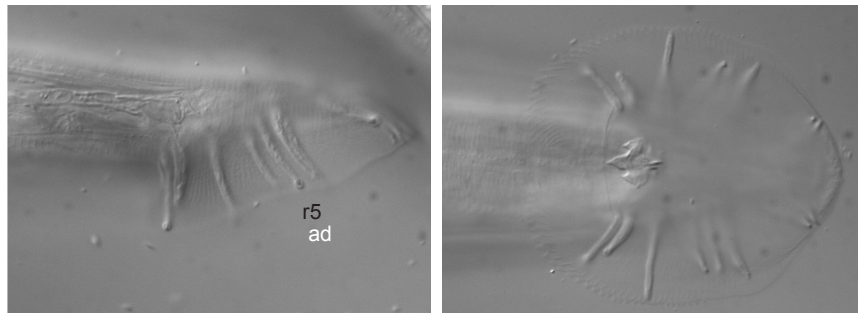

*C. ceno* HPT43

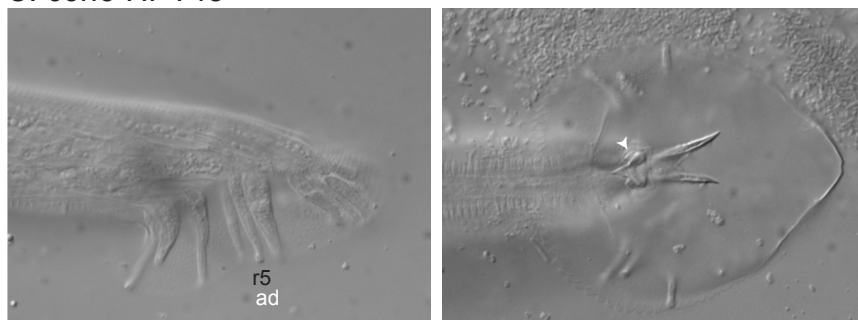

*C. brawijaya* HPT50

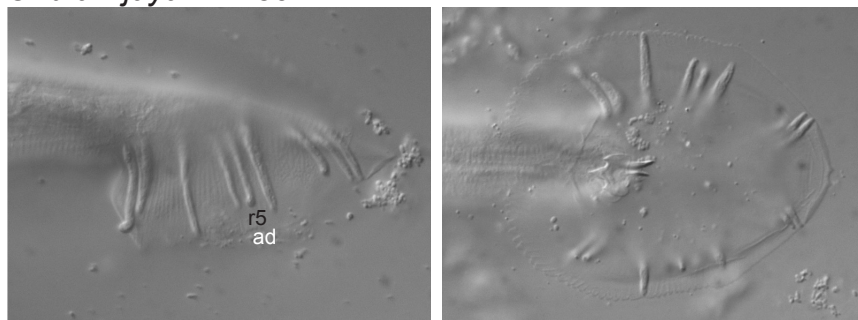

*C. ubi* HPT5

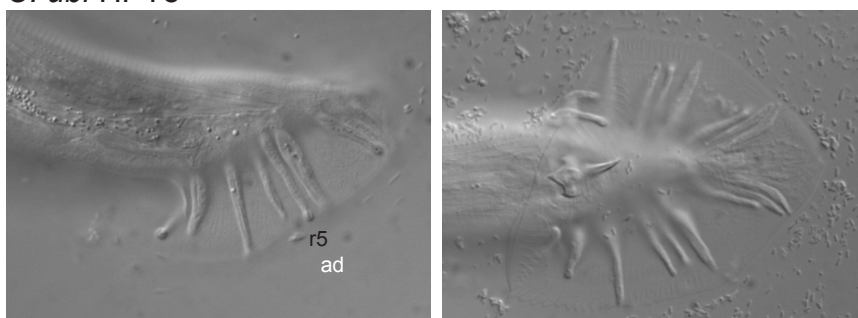

Figure S2

Supplement: Supplement 2 — Figure S2. Male tail micrographs of the five newly described species. Nomarski micrographs of individuals of the reference strain. Left: left lateral view. Right: Ventral view. Two different nomenclatures for tail sensory organs are used, one counting nine rays from anterior to posterior, the other distinguishing the ventral from the dorsal ones. In all species, the anterior dorsal ray (ad) is the fifth and the posterior dorsal ray (pd) is the seventh. The precloacal sensillum has a hook shape in all species, and is trilobed at least in all four species of the Sinica subclade. We note here that the lateral lobes of this precloacal sensory organ appear to be slightly more dorsal than the central part and could derive from fusion of the cuticle (dorso)-lateral to the central part of the hook (see arrowhead on the C. ceno HPT43 ventral view, for which the focal plane was chosen to demonstrate this). We further note that C. indonesiana HPT10, outside of the Sinica subclade, has a hint of a trilobed hook as well, with the cuticle dorso-lateral to the central part of the hook tending to attach to it. Bar: 10 μm, valid for all panels. [file media-2.pdf]
